# A CpG-methylation-based assay to predict survival in clear cell renal cell carcinoma

**DOI:** 10.1038/ncomms9699

**Published:** 2015-10-30

**Authors:** Jin-Huan Wei, Ahmed Haddad, Kai-Jie Wu, Hong-Wei Zhao, Payal Kapur, Zhi-Ling Zhang, Liang-Yun Zhao, Zhen-Hua Chen, Yun-Yun Zhou, Jian-Cheng Zhou, Bin Wang, Yan-Hong Yu, Mu-Yan Cai, Dan Xie, Bing Liao, Cai-Xia Li, Pei-Xing Li, Zong-Ren Wang, Fang-Jian Zhou, Lei Shi, Qing-Zuo Liu, Zhen-Li Gao, Da-Lin He, Wei Chen, Jer-Tsong Hsieh, Quan-Zhen Li, Vitaly Margulis, Jun-Hang Luo

**Affiliations:** 1 Department of Urology, First Affiliated Hospital, Sun Yat-sen University, No. 58, ZhongShan Second Road, Guangdong 510080, China; 2 Department of Urology, University of Texas Southwestern Medical Center at Dallas, Dallas, Texas 75390, USA; 3 Department of Urology, First Affiliated Hospital of Xi'an Jiaotong University, Shaanxi 710061, China; 4 Department of Urology, Affiliated Yantai Yuhuangding Hospital, Qingdao University Medical College, Shandong 264000, China; 5 Department of Pathology, University of Texas Southwestern Medical Center at Dallas, Dallas, Texas 75390, USA; 6 Department of Urology, Cancer Center, Sun Yat-sen University, Guangdong 510060, China; 7 Department of Urology, Affiliated Hospital of Kunming University of Science and Technology, Yunnan 650032, China; 8 Quantitive Biomedical Research Center, University of Texas Southwestern Medical Center at Dallas, Dallas, Texas 75390, USA; 9 Department of Pathology, Cancer Center, Sun Yat-sen University, Guangdong 510060, China; 10 Department of Pathology, First Affiliated Hospital, Sun Yat-sen University, Guangdong 510080, China; 11 School of Mathematics and Computational Science, Sun Yat-sen University, Guangdong 510275, China; 12 Department of Immunology and Microarray Core, University of Texas Southwestern Medical Center at Dallas, Dallas, Texas 75390, USA

## Abstract

Clear cell renal cell carcinomas (ccRCCs) display divergent clinical behaviours. Molecular markers might improve risk stratification of ccRCC. Here we use, based on genome-wide CpG methylation profiling, a LASSO model to develop a five-CpG-based assay for ccRCC prognosis that can be used with formalin-fixed paraffin-embedded specimens. The five-CpG-based classifier was validated in three independent sets from China, United States and the Cancer Genome Atlas data set. The classifier predicts the overall survival of ccRCC patients (hazard ratio=2.96−4.82; *P*=3.9 × 10^−6^−2.2 × 10^−9^), independent of standard clinical prognostic factors. The five-CpG-based classifier successfully categorizes patients into high-risk and low-risk groups, with significant differences of clinical outcome in respective clinical stages and individual ‘stage, size, grade and necrosis' scores. Moreover, methylation at the five CpGs correlates with expression of five genes: *PITX1*, *FOXE3*, *TWF2*, *EHBP1L1* and *RIN1*. Our five-CpG-based classifier is a practical and reliable prognostic tool for ccRCC that can add prognostic value to the staging system.

## Abstract (web summary)

Using molecular markers is a useful way to predict the prognosis of cancer patients. Here, Wei *et al.* describe a five gene methylation signature that can predict the prognosis of renal clear cell cancer and validate its use in multiple patient cohorts.

## Graphical abstract

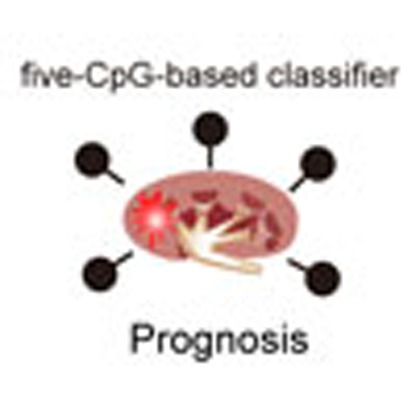

Renal cell carcinoma (RCC) is the most common malignant neoplasm arising from the kidney and it represents ∼2–3% of all human malignancies. The major histological subtype is clear cell RCC (ccRCC), accounting for 80–90% of all RCC cases^1^. TNM stage and Fuhrman grade remain the most commonly used predictors of clinical outcome for patients with ccRCC. Clinically integrated systems, such as the Mayo Clinic stage, size, grade and necrosis (SSIGN) score and the University of California Integrated Staging System, can improve prognostic accuracy^2^,^3^. However, patients with similar clinical features or integrated systems score may have diverse outcomes. Thus, there is a need to add prognostic value to the current staging system, which could be achieved with the use of validated biomarkers. Nevertheless, despite numerous studies, no reliable prognostic biomarkers for ccRCC have been identified or used routinely in clinical practice to date.

As DNA methylation is a crucial factor for cancer formation, it rapidly gained clinical attention as a biomarker for diagnosis and prognosis^4^,^5^,^6^. DNA methylation almost exclusively occurs at the C-5 position of cytosines in the sequence context of 5′-CpG-3′ in mammalian cells. As genome-wide technologies continue to develop, such as the development of the Infinium HumanMethylation27 array and HumanMethylation450 array, the understanding of CpG methylation associated with human cancers including RCC continues to rapidly improve^7^,^8^,^9^,^10^,^11^,^12^.

Here we develop and validate a practical and reliable classifier based on genome-wide CpG methylation profiling that improves risk stratification for patients with ccRCC. Moreover, we use the Cancer Genome Atlas (TCGA) data set to validate our prognostic classifier, investigate the relationship between CpG methylation and gene expression, and analyse the gene interaction network.

## Results

### Identifying candidate CpGs based on genome-wide profiling

**Figure 1:**
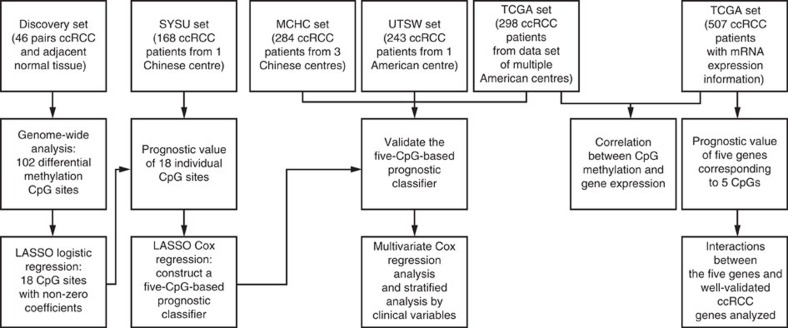
Flow chart indicating study design. We identified candidate CpGs sites from 46 paired ccRCC and adjacent normal tissues by CpG methylation microarray in the discovery set. We then used a multivariate LASSO Cox regression model to build a CpG-based prognostic classifier in SYSU set. Furthermore, the five-CpG-based classifier was validated in MCHC, UTSW and TCGA data sets. Relationship between CpG methylation, gene expression and patient prognosis were also analysed in the TCGA set.

**Figure 2:**
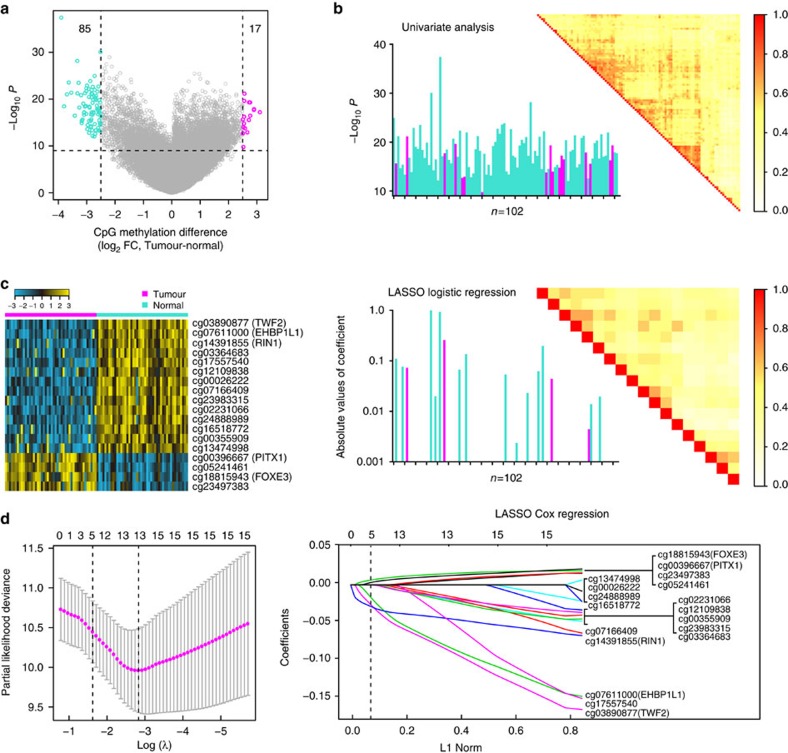
Construction of the five-CpG-based classifier. (**a**) One hundred and two CpG sites selected by univariate analysis. Volcano plot showing a comparison of CpG methylation for ccRCC tumour tissues versus adjacent normal tissues (*n*=46, HumanMethylation450 platform). This plot depicts the biological significance (log_2_ fold change (FC)) on the *X* axis and the statistical significance (−log_10_ *P*) on the *Y* axis. Log_2_ FC>2·5 for 102 CpGs; the methylation level of 17 CpGs is higher in tumour in comparison with normal tissue (magenta) and lower in 85 CpGs (turquoise). (**b**) Eighteen CpG sites selected by LASSO logistic regression analysis. Histogram of the univariate *t*-test *P*-values is shown, in the upper left panel, as −log_10_ *P* for all 102 CpGs. A matrix representing the pairwise correlation (*r*^2^, Spearman's correlation) between the CpGs is displayed in the upper right panel. The lower left panel shows a histogram of the absolute values of the coefficients for all 102 CpGs, of which 18 had non-zero coefficients by LASSO logistic regression analysis. The correlation structure between the 18 CpGs with non-zero coefficients is shown in the lower right panel, demonstrating reduced multicollinearity. (**c**) Heatmap showing methylation of the 18 CpGs in ccRCC tumour tissue (46 samples) and adjacent normal tissue (46 samples). (**d**) Five CpG sites selected by LASSO Cox regression analysis. Left panel: the two dotted vertical lines are drawn at the optimal values by minimum criteria (right) and 1−s.e. criteria (left). Details are provided in Methods. Right panel: LASSO coefficient profiles of the 18 CpGs. A vertical line is drawn at the optimal value by 1−s.e. criteria and results in five non-zero coefficients. Five CpGs—cg00396667 (*PITX1*), cg18815943 (*FOXE3*), cg03890877 (*TWF2*), cg07611000 (*EHBP1L1*) and cg14391855 (*RIN1*)—with coefficients 0.0066, 0.0034, −0.027, −0.018 and −0.03, respectively, were selected in the LASSO Cox regression model.

We analysed 46 paired ccRCC and adjacent normal tissues by CpG methylation microarray (Infinium HumanMethylation450 array) in the discovery set (Supplementary Table 1) and looked for differential methylation in ccRCC tumours and normal tissue at CpG sites across the genome (Fig. 1). The volcano plot (Fig. 2a) showed that the log_2_ fold change of 102 CpG sites was more than 2.5 for 46 pairs of tumour and adjacent normal tissue, based on the genome-wide analysis of CpG methylation (*t*-test, all *P*<10^−9^; false discovery rate <10^−8^; Supplementary Data 1). The 102 CpGs identified in univariate analysis were entered into a multivariate logistic regression model (the least absolute shrinkage and selection operator (LASSO)) and 18 had non-zero coefficients (Fig. 2b,c).

### Constructing and validating the CpG-based classifier

**Figure 3:**
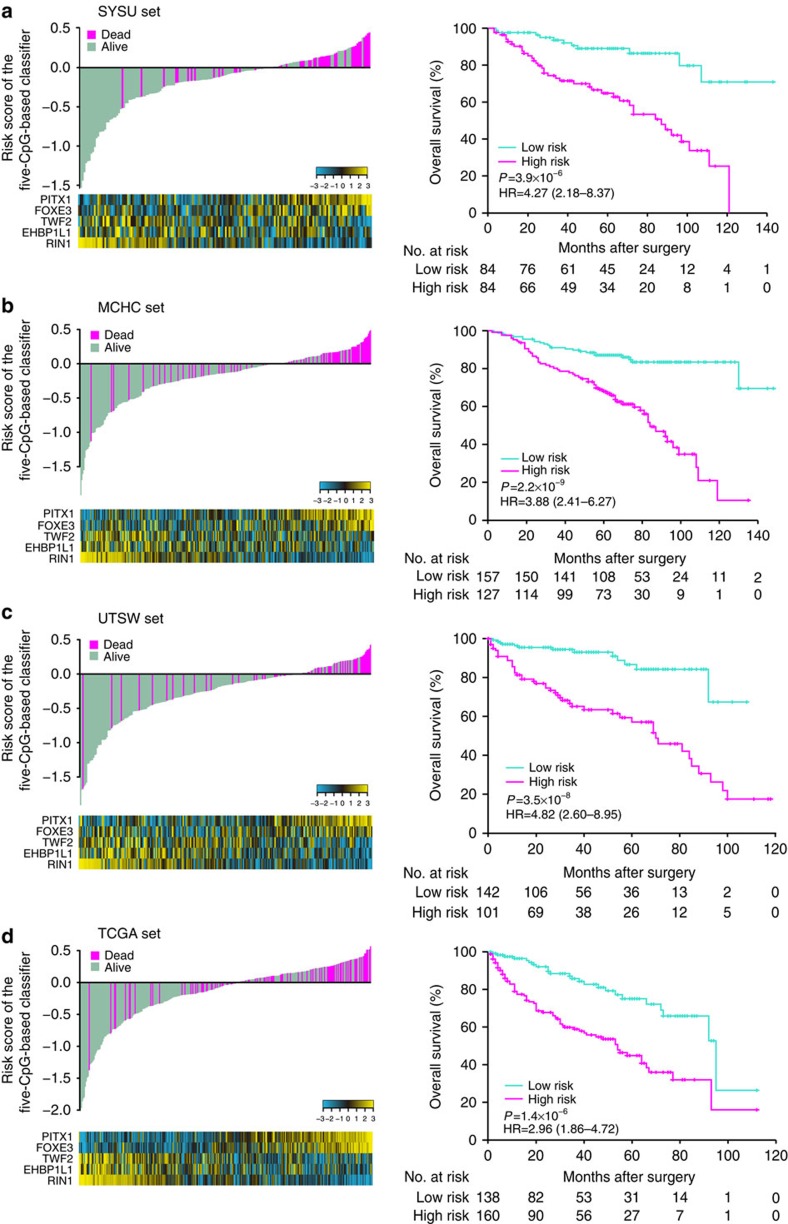
Risk score calculated by the five-CpG-based classifier and Kaplan–Meier survival in the four different sets. (**a**) SYSU set, (**b**) MCHC set, (**c**) UTSW set and (**d**) TCGA set. Upper left panel: risk-score distribution of the five-CpG-based classifier and patient survival status. Lower left panel: heatmap showing methylation of the five CpGs in the patients. Right panel: Kaplan–Meier survival analysis for the patients. The patients were divided into low-risk and high-risk groups using the median cutoff value of the classifier risk score (−0.1). *P*-values were calculated using the log-rank test. HR, hazard ratio.

We then carried out pyrosequencing to quantify the methylation value of these 18 CpG sites by using formalin-fixed, paraffin-embedded (FFPE) specimens from the Sun Yat-sen University (SYSU) set of 168 ccRCC patients. Supplementary Table 3 shows univariate Cox regression analysis of overall survival based on each of the 18 CpGs in the SYSU set (*P*=0.49–0.001). We used a multivariate LASSO Cox regression model to build a CpG-based prognostic classifier, which included 5 of the 18 CpGs: cg00396667, cg18815943, cg03890877, cg07611000 and cg14391855 (Fig. 2d and Supplementary Fig. 1). These five CpG sites were in the regions of genes *PITX1*, *FOXE3*, *TWF2*, *EHBP1L1* and *RIN1*, respectively. Using the LASSO Cox regression models, we also calculated a risk score for each patient based on individualized values of methylation for the five genes: risk score=(0.0066 × PITX1)+(0.0034 × FOXE3)−(0.027 × TWF2)−(0.018 × EHBP1L1)−(0.03 × RIN1). When we assessed the distribution of risk scores for the five-CpG-based classifier and survival status, patients with lower risk scores generally had better survival than those with higher risk scores (Fig. 3a, left panel). Patients in the SYSU set were divided into high-risk or low-risk groups, using the median risk score (−0.1) as the cutoff. Compared with patients in low-risk group, patients in the high-risk group had shorter overall survival (hazard ratio=4.27, 95% confidence interval=2.18–8.37, log-rank test *P*=3.9 × 10^−6^; Fig. 3a, right panel).

To estimate the reproducibility and validity of the five-CpG-based classifier, we performed international validation using data sets comprising ccRCC patients from a site in the United States (University of Texas Southwestern Medical Center at Dallas, UTSW set, 243 cases) and multiple clinical centres in China (MCHC set, 284 cases). Furthermore, we used the external data set, TCGA data set (298 cases), to validate our five-CpG-based classifier (Fig. 1 and Table 1). Methylation value of the five CpG sites is shown for each set in Supplementary Fig. 2. The risk score for each patient in the sets was calculated with the same formula used in the SYSU set, patients with lower risk scores generally had better survival than those with higher risk scores (Fig. 3b–d, left panel). Patients in these three sets were classified into high-risk and low-risk groups with the same cutoff used in the SYSU set (−0.1). Patients in the high-risk groups had shorter overall survival than those in the low-risk groups in all three sets (hazard ratio=2.96–4.82, log-rank test *P*=1.4 × 10^−6^–2.2 × 10^−9^; Fig. 3b–d (right panel) and Supplementary Table 4). After adjusting for standard clinical prognostic factors (age, TNM stage, Fuhrman grade and necrosis status), the five-CpG-based classifier remained an independent prognostic factor in the SYSU set and the three other patient sets (Table 2, all *P*<0.05).

### Stratification analysis of the five-CpG-based classifier

**Figure 4:**
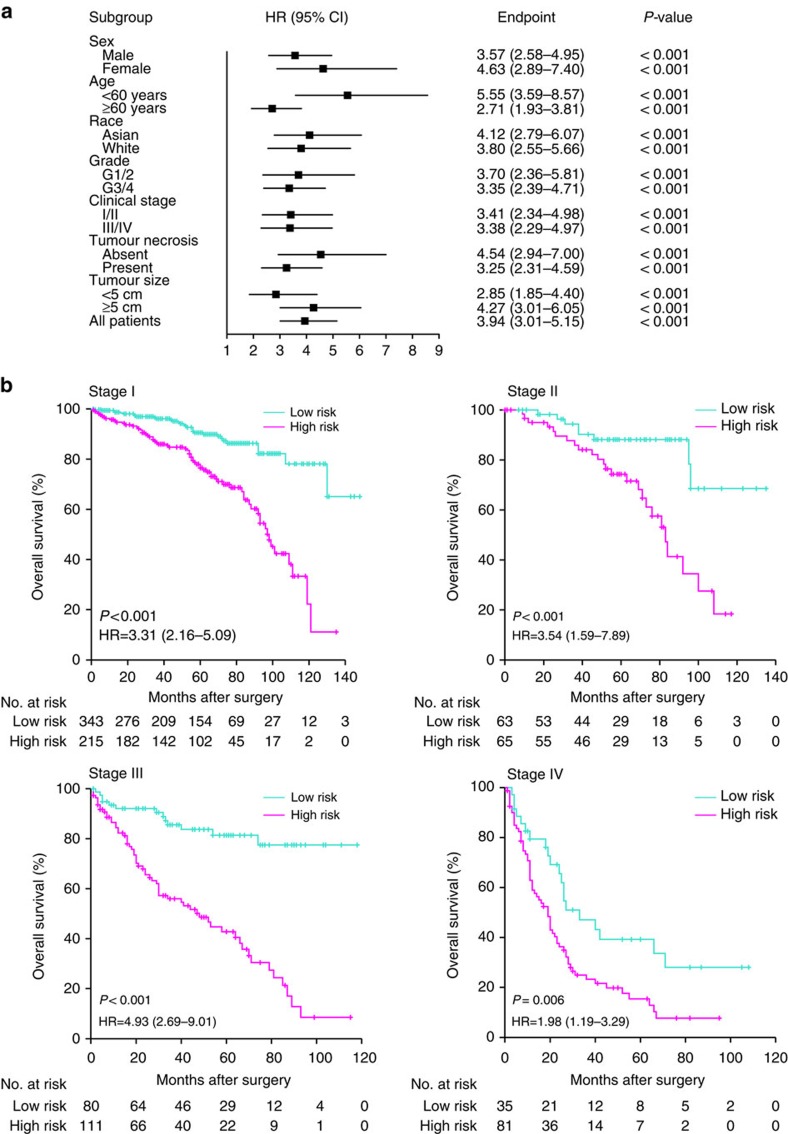
Stratification analysis of the five-CpG-based classifier. (**a**) Hazard ratio (HR) of overall mortality for all 993 patients with ccRCC according to the five-CpG-based classifier in different subgroups stratified by clinical parameters. (**b**) Kaplan–Meier survival analysis of the five-CpG-based classifier in subsets of different clinical stage patients with ccRCC (log-rank test).

Survival analysis was further performed with regard to the five-CpG-based classifier in subsets of patients with different clinical variables. When stratified by clinical variables (sex, age, race, Fuhrman grade, tumour size and necrosis status), the five-CpG-based classifier was still a clinically and statistically significant prognostic model (Fig. 4a, Supplementary Fig. 3 and Supplementary Table 5). As shown in Fig. 4b, the ccRCC patients in the same clinical stage could be successfully separated into the subgroups of better prognosis and poorer prognosis by the five-CpG-based classifier (log-rank test, all *P*<0.05).

**Figure 5:**
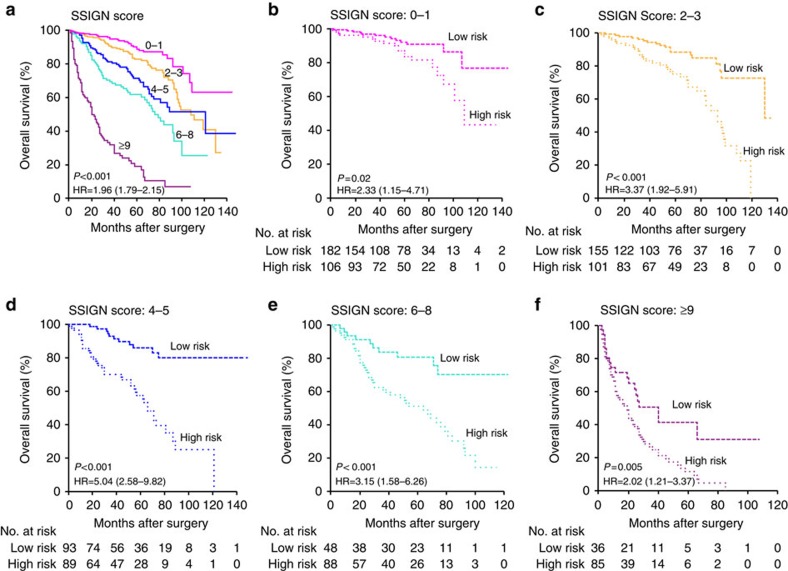
Analysis of the five-CpG-based classifier in subsets of different SSIGN-score categories. (**a**)The Kaplan–Meier curves regarding overall survival for respective SSIGN-score categories. (**b**–**f**) Kaplan–Meier survival analysis of the five-CpG-based classifier in subsets of different SSIGN-score categories (log-rank test). HR, hazard ratio.

The SSIGN score (ranging from 0 to 15) is one of the clinically integrated systems that was introduced to improve prognostic accuracy in ccRCC (Supplementary Table 6). The Kaplan–Meier curves regarding overall survival for respective SSIGN-score categories are shown in Fig. 5a. The five-CpG-based classifier successfully categorized patients into high-risk and low-risk groups with significant differences of clinical outcome in each of the SSIGN-score categories (log-rank test, all *P*<0.05; Fig. 5b-f). Thus, the five-CpG-based classifier can add prognostic value to both the clinical stage and the SSIGN score.

### Impact of intratumour heterogeneity

To determine whether intratumour heterogeneity (ITH) affected risk score and risk stratification based on the five-CpG-based classifier, we assayed methylation value of the five CpG sites in three different regions within 23 ccRCC tumours. As shown in Supplementary Fig. 5, inter-individual differences in the methylation of the five CpG sites, assessed by averaging all measurements from the same tumour, were significantly higher than measurement differences within individual tumours. ITH had an obviously smaller effect on classifier-based risk scores (coefficient of variation (CV), 10.5%) than on the five individual CpGs (CV, 15.2–22.3%). ITH affected risk stratification in 2 (8.7%) of the 23 tumours, suggesting the 5-CpG-based classifier is a precise tool (Supplementary Table 7).

### CpG methylation and gene expression and patient prognosis

Using the TCGA data set, we analysed whether methylation of the five CpGs was correlated with gene expression, as per Spearman's correlation. We observed that the correlation between methylation value and gene expression by Spearman's correlation test was significantly inverse for *TWF2* (*P*=5.8 × 10^−11^), *EHBP1L1* (*P*=1.9 × 10^−6^) and *RIN1* (*P*=1.2 × 10^−30^), significantly positive for *PITX1* (*P*=4.1 × 10^−8^) and marginally positive for FOXE3 (*P*=0.09).

**Figure 6:**
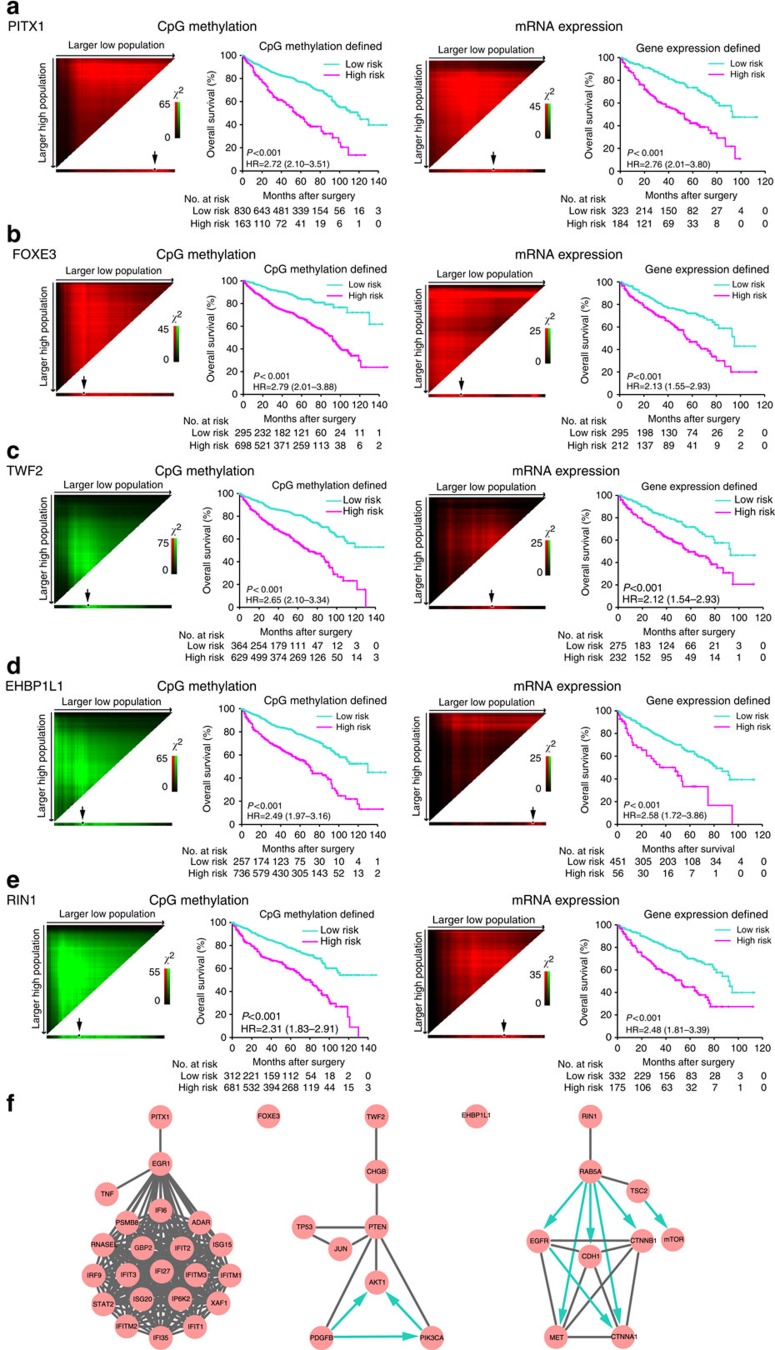
X-tile plots of the genes that correspond to the five CpGs and network analyses. X-tile plots of the CpG methylation (993 patients in the entire cohort) and mRNA expression of the five genes (507 patients in the TCGA data set): (**a**) *PITX1*, (**b**) *FOXE3*, (**c**) *TWF2*, (**d**) *EHBP1L1* and (**e**) *RIN1*. X-tile plots provide a single and intuitive method to assess the association between marker expression and survival, and automatically select the optimum cut point according to the highest *χ*^2^-value defined by Kaplan–Meier survival analysis and log-rank test. Colouration of the plot represents the strength of the association at each division, ranging from low (dark, black) to high (bright, red or green). Red represents inverse association between marker expression and survival, whereas green represents direct association between marker expression and survival. Each pixel represents an individual cutpoint where the number of patients in the group increases as progressed down for the high-expression group (‘larger high population') or to the right for the low-expression group (‘larger low population'). The dark dots (indicated by arrow) in the X-tile plots are the sites according to the highest *χ*^2^-value and are used as the cutoff points separating patients into high-risk and low-risk groups. (**f**) Network analyses of the genes that correspond to the five CpGs by cBioPortal. *PITX1*, *TWF2* and *RIN1* were predicted to have an impact on a diverse network of genes and pathways, as per the cBioPortal for Cancer Genomics network analysis tool. Black line means interactions between the two entities; blue arrow represents that the first entity controls a reaction that changes the state of the second entity. HR, hazard ratio.

Nine hundred and ninety-three patients in the entire cohort were separated into CpG-defined high-risk and low-risk groups using X-tile plots, to generate the optimum cutoff score for methylation of the five CpGs. Kaplan–Meier survival analysis, depicted in Fig. 6a–e (left panel), showed the overall survival of patients in the CpG-defined low-risk group was significantly better than in the high-risk group. In addition, expression of the genes corresponding to the 5 CpGs effectively predicted the clinical outcome of the 507 patients for whom there were messenger RNA expression data in the TCGA data set (Fig. 6a–e, right panel).

### Integrating our results with genes linked to RCC

To further evaluate the role of genes corresponding to the five CpGs in relation to well-validated ccRCC susceptibility genes, we used the cBioPortal for Cancer Genomics network to evaluate gene connectivity. As shown in Fig. 6f, *PITX1* interacts with *EGR1*, which is then connected to an immune response network. *RIN1* interacts with *RAB5A*, which is connected to genes that are involved in cancer cell epithelial-to-mesenchymal transition. *TWF2* mainly participates in cancer cell proliferation signalling pathways through interaction with chromogranin B (*CHGB*). *FOXE3* and *EHBP1L1* showed exceptionally low connectivity in the database.

## Discussion

Integrating multiple biomarkers into a single model would substantially improve prognostic value compared with a single biomarker^13^. As genome-wide technologies have become more sophisticated, so too have molecular prognostic models, which can now integrate mRNA, microRNA, CpG and single-nucleotide polymorphism (SNP) data^7^,^14^,^15^,^16^,^17^,^18^,^19^. However, early studies with integrated models had several notable limitations. (1) There was a lack of information (such as risk score formulas or biomarker coefficients) on how to integrate multiple biomarkers into one model, which restricted wide use of these models in the clinic. (2) Some models incorporated too many biomarkers, making it nearly impossible to apply them in clinical practice. (3) Inappropriate statistical methods were used to mine microarray data. More specifically, in microarray analysis, the number of covariates is usually close to or larger than the number of observations. The Cox proportional hazards regression analysis, which is the most popular approach for modelling covariate information for survival times, is unsuitable for high-dimensional microarray data when the sample-size-to-variables ratio is too low (such as <10:1)^20^,^21^. The LASSO model used in our study is one of the statistical methods that can eliminate this limitation^22^,^23^,^24^. (4) Models were developed based on analysis of fresh-frozen specimens, limiting immediate clinical application in a broad community setting. (5) Models were not validated in multiple independent cohorts. Thus, none of the integrated prognostic models developed using genome-wide, microarray-based analysis are being used in clinical practice. In this study, we developed a practical CpG-methylation-based assay that can be used with FFPE material to identify prognostic CpG information and demonstrated how this information can be integrated into a prognostic model that is feasible to use in the clinic.

ITH can impair the precise molecular analysis of tumours, because biomarker expression can vary across different tumour regions^25^. Some prognostic biomarkers could not be validated in previous reports and one possible cause was large intra-sample variability in gene expression^26^. However, two recent studies showed ITH, although present at the level of individual gene expression, did not preclude precise microarray-based predictions of clinical outcome in ccRCC or breast cancer^26^,^27^. Compared with a single prognostic biomarker, our integrated prognostic models based on microarray profiling not only have higher prognostic accuracy but also are less influenced by ITH.

Several studies have analysed gene expression profiles in RCC and examined their potential clinical relevance^28^,^29^,^30^,^31^. These signatures contained large numbers of genes that were detected by microarray or reverse transcriptase–PCR and, consequently, these signatures had limited use in clinical practice. In this study, we identified methylation level of five highly prognostic CpG sites by pyrosequencing from the FFPE material. Given the fewer number of markers, our classifier is both more feasible and cheaper compared with the prognostic signatures proposed in previous studies. The five-CpG-based classifier can accurately distinguish between patients with ccRCC, with substantially different clinical outcomes, even after adjustment for standard clinical prognostic factors, such as age, TNM stage, Fuhrman grade and necrosis status. We further performed international validation using data sets comprising patients from a site in the United States and MCHC, as well as patients in TCGA data set, who were also from multiple centres in the United States. The prognostic accuracy of the five-CpG-based classifier was similar in the three validation sets. The classifier was reproducible regardless of clinical centre, country or race and it can provide prognostic value that complements the clinical stage and the SSIGN score.

Five genes corresponded to the five CpGs identified in our study: *FOXE3*, *PITX1*, *RIN1*, *TWF2* and *EHBP1L1*. DNA methylation of *FOXE3* has been reported and validated as a diagnostic biomarker for paediatric acute lymphoblastic leukemia^32^. Hypermethylation of *PITX1* and *RIN1* has been described in human salivary gland adenoid cystic carcinoma and breast cancer, respectively^33^,^34^. *TWF2* has been implicated in neurite outgrowth^35^. However, the function of *EHBP1L1* remains unknown. Our pathway analysis results showed that these genes may play diverse roles in regulating ccRCC progression, including tumour immune response, cancer cell proliferation and epithelial-to-mesenchymal transition. Notably, these genes are all distributed at the periphery of the signalling network, in contrast to central network markers such as *PTEN* and *TP53*. This finding is similar to recent studies showing that epigenetic marker drift occurs preferentially in genes that occupy peripheral network positions of exceptionally low connectivity^7^,^36^,^37^.

In conclusion, the present study suggests the newly developed five-CpG-based classifier is a practical and powerful prognostic tool for ccRCC, which can provide prognostic value that complements the current staging system of ccRCC and will facilitate patient counselling, tailoring of follow-up protocols and selection for appropriate adjuvant trial designs.

## Methods

### Patients

In this study, we used 695 FFPE tissue samples from 695 patients who underwent resection of a ccRCC. The SYSU set included 168 patients from the First Affiliated Hospital and Cancer Center of SYSU (Guangdong, Southeast China) treated between 2001 and 2009. The MCHC set included 284 patients treated between 2001 and 2009 at three hospitals across different regions of China: First Affiliated Hospital of Xi'an Jiaotong University (Shaanxi, Northwest China), Affiliated Yantai Yuhuangding Hospital of Qingdao University Medical College (Shandong, Northeast China) and Affiliated Hospital of Kunming University of Science and Technology (Yunnan, Southwest China) between 2001 and 2009. Another 243 patients from the University of Texas Southwestern Medical Center at Dallas (TX, USA) treated between 2004 and 2011 comprised the UTSW set. The TNM 2009 staging system was used to classify ccRCC patients. The grading system used in the study was based on the Fuhrman four grade. Clinical baseline data were obtained through medical record review. Patients with sporadic, unilateral ccRCC and with clinicopathological characteristics and follow-up information available were included. In addition, to generate CpG methylation expression profiles we obtained, as a discovery set, a panel of 46 fresh-frozen tumour samples with paired adjacent normal tissue from patients with ccRCC treated between 2011 and 2013 at the First Affiliated Hospital of SYSU. Consent was obtained for all subjects and the protocols approved by the respective Institutional Review Board of each institution.

### Infinium methylation assay microarrays

In the discovery set, we used the HumanMethylation450 BeadChip (Illumina, San Diego, CA, USA) for genome-wide assessment of methylation at CpG sites^38^. Genomic DNA was extracted from 46 paired ccRCC tumour and adjacent normal tissues with the QIAamp DNA mini kit (Qiagen, Valencia, CA, USA) following the manufacturer's recommendations. All DNA samples were assessed for integrity, quantity and purity by electrophoresis in a 1.3% agarose gel, PicoGreen quantification and NanoDrop measurements, respectively. The samples that passed quality control were processed with Infinium HumanMethylation450 BeadChip Kits (Illumina) according to the manufacturer's recommendations, through automated processes in the Genomic and Microarray Core, University of Texas Southwestern Medical Center. Arrays were imaged with BeadArray Reader using standard Illumina scanner settings. The signal data were extracted and processed using RnBeads^39^ version 0.99.12 in the R software 3.0.3. We considered a methylation *β*-value to be unreliable if its corresponding detection *P*-value was not below the threshold *T*=0.05. Both sites and samples were filtered using a greedy approach. BMIQ normalization methods and the background subtraction ‘methylumi.noob' methods implemented in the RnBeads package was applied^40^,^41^. We removed probes containing an SNP in the assayed CpG dinucleotide, as well as those for which two or more SNPs were located in the probe sequence^7^. We removed probes not mapping uniquely to the human reference genome (hg19) allowing for one mismatch under the criteria of Price *et al.*^42^ Non-CpG targeting probes (Ch probes) and the probes included in the sex chromosomes were also removed^43^. Using the annotations provided by Illumina for the HumanMethylation450 platform, only probes located in the CpG islands and shores were kept for analysis in this study. The R Linear Models for Microarray Data (Limma) package^44^ was used to compare *β*-values and to identify differentially methylated probes between cancer and adjacent normal tissues. *P*-values were calculated from the moderated *t*-statistics and multiple testing correction of the *P*-values was performed using Benjamini and Hochberg's method (false discovery rate), to identify differentially methylated probes. Microarray data were uploaded to the National Center for Biotechnology Information's Gene Expression Omnibus (Series GSE61441, http://www.ncbi.nlm.nih.gov/geo/query/acc.cgi?token=ufaxumuubrqxpgr&acc=GSE61441).

### Pyrosequencing

The methylation level of CpG sites was evaluated with pyrosequencing in the SYSU, MCHC and UTSW sets. DNA from paraffin-embedded tissue blocks was extracted from four sequential unstained sections, each 15 μm thick. For each sample of tumour tissue, subsequent sections were stained with haematoxylin and eosin for histological confirmation of the presence (>70%) of tumour cells. Genomic DNA was extracted with the QIAamp DNA FFPE Tissue Kit (Qiagen) following the manufacturer's recommendations. Bisulfite conversion was performed on 1 μg of DNA with the EpiTect Bisulfite Kit (Qiagen). Twenty nanograms of converted DNA was used as a template in each subsequent PCR. Specific sets of primers for PCR amplification and sequencing were designed using the PyroMark Assay Design 2.0 software (Qiagen). All primer sequences are listed in Supplementary Table 2. PCRs were performed with the PyroMark PCR Kit (Qiagen) under the following conditions: 95 °C for 15 min, 45 cycles of 94 °C for 30 s, 56 °C for 30 s and 72 °C for 30 s, and an elongation step of 72 °C for 10 min. The success of amplification was assessed by 2% agarose gel electrophoresis. PCR products were pyrosequenced with the PyroMark Q24 pyrosequencer (Qiagen) according to the manufacturer's protocol (Pyro-Gold reagents). Output data were analysed using PyroMark Q24 2.0.6 Software (Qiagen), which calculates the CpG methylation value as the percentage (mC/[mC+C]) for each CpG site, allowing quantitative comparisons. Controls to assess proper bisulfite conversion of the DNA were included in each run and sequencing controls were used to ensure the fidelity of the measurements.

### TCGA data and network analysis

For the TCGA set, clinical data, CpG methylation value (level 3 data, Infinium HumanMethylation450) and mRNA expression (level 3 data, RNA-seq Version 2 Illumina) were downloaded from the TCGA data portal (http://tcga-data.nci.nih.gov/tcga/) on 1 October 2014. The clinical data included 512 retrospectively identified patients who underwent radical or partial nephrectomy between 1998 and 2010 for sporadic ccRCC^45^. Of the 512 patients, CpG methylation data were available for 298 patients and mRNA expression data were available for 507 patients. Of the 298 patients, *VHL*, *PBRM1* and *BAP1* gene mutation data were available for 242 (Supplementary Fig. 6). The cBioPortal for Cancer Genomics (http://cbioportal.org) network was used to search for pathways and interactions that might be linked to genes that correspond to the identified CpG sites in ccRCC^46^.

### Intratumour heterogeneity

ITH was investigated by extracting DNA samples from morphologically distinct regions within the tumours of 23 patients with ccRCC treated between 2011 and 2013 at the First Affiliated Hospital of SYSU (FFPE specimens; three different regions coded as R1, R2 and R3; Supplementary Fig.4). Methylation of the five CpG sites was detected with pyrosequencing. The s.d. and CV were used to describe the inter-sample variability of CpG methylation between the 23 ccRCCs and the intra-sample variability between different regions.

### Statistical analysis

The goal of this study was to identify prognostic classifier that predicts overall survival. This is defined as the time between surgery and death or the last follow-up date. Volcano plot analysis was used to select CpG sites based on absolute fold change in combination with *t*-test *P*-values. LASSO logistic regression analysis was used to identify the candidate CpG sites with non-zero coefficients in the discovery set. LASSO Cox regression analysis was used to select the prognostic markers of the candidate CpG sites and to construct a multi-CpG-based classifier for predicting the overall survival of patients with ccRCC in the SYSU set. We used the Kaplan–Meier method to analyse the correlation between variables and overall survival, and we used the log-rank test to compare survival curves. Multivariate survival analysis was performed using the Cox regression model. X-tile plots were used to generate the optimum cutoff point for continuous variables according to the highest *χ*^2^-value defined by Kaplan–Meier survival analysis and log-rank test^47^. X-tile plots were created with X-tile software version 3.6.1 (Yale University School of Medicine, New Haven, CT, USA) and all the other statistical tests were performed with R software version 3.0.3 (R Foundation for Statistical Computing, Vienna, Austria). Statistical significance was set at 0.05.

### LASSO regression analysis

The high dimensionality of microarray-based experiments in contrast to the small number of samples easily leads to overfitting. Regularized linear models such as logistic regression with LASSO penalty are popular solutions to fitting sparse models in which only a small subset of features plays a role^48^. LASSO can be used with high-dimensional data for optimal selection of genes with a strong diagnostic or prognostic value and low correlation among each other to prevent overfitting^49^,^50^,^51^,^52^. LASSO is a form of regularized or ‘penalized' regression where L1 regularization is introduced into the standard multiple linear regression procedure using a compound cost function to optimize the regression coefficients. LASSO regression shrinks the coefficient estimates towards zero, with the degree of shrinkage depending on an additional parameter, *λ*. In this way, coefficient estimates can be forced to be exactly zero, thereby effectively eliminating a number of variables. We adopted the LASSO regression model to achieve shrinkage and variable selection simultaneously. Ten-time cross-validations were used to determine the optimal values of *λ* (refs 51, 52, 53). We choose *λ* via 1−s.e. criteria, that is, the optimal *λ* is the largest value for which the partial likelihood deviance is within 1 s.e. of the smallest value of partial likelihood deviance^24^. We used R software version 3.0.3 (R Foundation for Statistical Computing) and the ‘glmnet' package to perform LASSO regression analysis.

## Additional information

**Accession codes**: Methylation array data have been deposited in Gene Expression Omnibus database under accession code GSE61441.

**How to cite this article:** Wei, J.-H. *et al.* A CpG-methylation-based assay to predict survival in clear cell renal cell carcinoma. *Nat. Commun.* 6:8699 doi: 10.1038/ncomms9699 (2015).

## Supplementary Material

Supplementary Information Supplementary Figures 1-6 and Supplementary Tables 1-7

Supplementary Data 1 Supplementary Data 1: 102 highly ranked differentially methylated CpGs in 46 paired tumor and adjacent normal tissues of ccRCC.

## Figures and Tables

**Table 1:** Baseline characteristics of patients by the five-CpG-based classifier assessment set.

**Characteristic**	**SYSU set (*n*=168)**			**MCHC set (*n*=284)**			**UTSW set (*n*=243)**			**TCGA set (*n*=298)**		
	**No.of patients**	**Low risk (%)**	**High risk (%)**	**No.of patients**	**Low risk (%)**	**High risk (%)**	**No.of patients**	**Low risk (%)**	**High risk (%)**	**No.of patients**	**Low risk (%)**	**High risk (%)**
*Age (years)*												
<60	107	51 (48%)	56 (52%)	178	104 (58%)	74 (42%)	128	82 (64%)	46 (36%)	129	71 (55%)	58 (45%)
≥60	61	33 (54%)	28 (46%)	106	53 (50%)	53 (50%)	115	60 (52%)	55 (48%)	169	67 (40%)	102 (60%)
												
*Sex*												
Male	113	55 (49%)	58 (51%)	190	109 (57%)	81 (43%)	151	87 (58%)	64 (42%)	193	71 (37%)	122 (63%)
Female	55	29 (53%)	26 (47%)	94	48 (51%)	46 (49%)	92	55 (60%)	37 (40%)	105	67 (64%)	38 (36%)
												
*Race*												
Asian	168	84 (50%)	84 (50%)	284	157 (55%)	127 (45%)	4	1 (25%)	3 (75%)	1	0 (0%)	1 (100%)
White	0			0			183	104 (57%)	79 (43%)	264	120 (45%)	144 (55%)
Black	0			0			36	23 (64%)	13 (36%)	30	18 (60%)	12 (40%)
Not available	0			0			20	14 (70%)	6 (30%)	3	0 (0%)	3 (100%)
												
*Grade*												
G1	8	6 (75%)	2 (25%)	21	15 (71%)	6 (29%)	10	8 (80%)	2 (20%)	6	6 (100%)	0 (0%)
G2	87	42 (48%)	45 (52%)	134	80 (60%)	54 (40%)	128	84 (66%)	44 (34%)	123	75 (61%)	48 (39%)
G3	51	25 (49%)	26 (51%)	88	45 (51%)	43 (49%)	77	38 (49%)	39 (51%)	120	50 (42%)	70 (58%)
G4	22	11 (50%)	11 (50%)	41	17 (41%)	24 (59%)	28	12 (43%)	16 (57%)	49	7 (14%)	42 (86%)
												
*Tumour size*												
**<**5 cm	60	33 (55%)	27 (45%)	140	76 (54%)	64 (46%)	136	93 (68%)	43 (32%)	119	76 (64%)	43 (36%)
≥5 cm	108	51 (47%)	57 (53%)	144	81 (56%)	63 (44%)	107	49 (46%)	58 (54%)	178	62 (35%)	116 (65%)
Not available	0			0			0			1	0 (0%)	1 (100%)
												
*Tumour necrosis*												
Absent	104	56 (54%)	48 (46%)	189	102 (54%)	87 (46%)	164	103 (63%)	61 (37%)	138	71 (51%)	67 (49%)
Present	64	28 (44%)	36 (56%)	95	55 (58%)	40 (42%)	70	32 (46%)	38 (54%)	160	67 (42%)	93 (58%)
Not available	0			0			9	7 (78%)	2 (22%)	0		
												
*pT*												
T1	97	49 (51%)	48 (49%)	180	101 (56%)	79 (44%)	156	107 (69%)	49 (31%)	145	95 (66%)	50 (34%)
T2	30	15 (50%)	15 (50%)	54	27 (50%)	27 (50%)	30	10 (33%)	20 (67%)	38	18 (47%)	20 (53%)
T3	37	17 (46%)	20 (54%)	46	27 (59%)	19 (41%)	52	24 (46%)	28 (54%)	107	23 (21%)	84 (79%)
T4	4	3 (75%)	1 (25%)	4	2 (50%)	2 (50%)	5	1 (20%)	4 (80%)	8	2 (25%)	6 (75%)
												
*pN*												
N0	152	78 (51%)	74 (49%)	267	151 (57%)	116 (43%)	226	134 (59%)	92 (41%)	129	62 (48%)	67 (52%)
N1	16	6 (37%)	10 (63%)	17	6 (35%)	11 (65%)	17	8 (47%)	9 (53%)	8	1 (12%)	7 (88%)
NX	0			0			0			161	75 (47%)	86 (53%)
												
*M*												
M0	163	83 (51%)	80 (49%)	274	150 (55%)	124 (45%)	221	136 (62%)	85 (38%)	244	125 (51%)	119 (49%)
M1	5	1 (20%)	4 (80%)	10	7 (70%)	3 (30%)	22	6 (27%)	16 (73%)	54	13 (24%)	41 (76%)
												
Stage (clinical)												
Stage I	91	45 (49%)	46 (51%)	171	96 (56%)	75 (44%)	155	107 (69%)	48 (31%)	141	95 (67%)	46 (33%)
Stage II	27	15 (56%)	12 (44%)	48	24 (50%)	24 (50%)	25	9 (36%)	16 (64%)	28	15 (54%)	13 (46%)
Stage III	36	17 (47%)	19 (53%)	43	28 (65%)	15 (35%)	39	20 (51%)	19 (49%)	73	15 (20%)	58 (80%)
Stage IV	14	7 (50%)	7 (50%)	22	9 (41%)	13 (59%)	24	6 (25%)	18 (75%)	56	13 (23%)	43 (77%)

MCHC, multiple clinical centres in China; SYSU, Sun Yat-sen University; TCGA, The Cancer Genome Atlas; UTSW, University of Texas Southwestern Medical Center at Dallas.

**Table 2:** Multivariate Cox regression analysis of the five-CpG-based classifier with overall survival in the four sets.

**Parameters**	**SYSU set**		**MCHC set**		**UTSW set**		**TCGA set**	
	**HR (95% CI)**	***P*****-value**	**HR (95% CI)**	***P*****-value**	**HR (95% CI)**	***P*****-value**	**HR (95% CI)**	***P*****-value**
Age (younger than 60 years versus 60 years or older)	1.18 (0.66–2.11)	0.58	2.13 (1.36–3.33)	0.001	1.76 (0.98–3.14)	0.06	1.28 (0.81–2.02)	0.29
pT (T1/2 versus T3/4)	2.82 (1.42–5.56)	0.003	1.99 (1.20–3.31)	0.008	2.39 (1.27–4.50)	0.007	1.63 (1.01–2.63)	0.05
pN (N0 versus N1)	3.16 (1.37–7.28)	0.007	4.59 (2.39–8.83)	<0.001	2.01 (0.95–4.26)	0.07	—*	—*
M (M0 versus M1)	7.41 (1.97–27.89)	0.003	1.61 (0.60–4.27)	0.34	3.10 (1.46–6.57)	0.003	2.77 (1.78–4.31)	<0.001
Grade (G1/2 versus G3/4)	1.88 (0.97–3.66)	0.06	1.60 (1.01–2.56)	0.05	1.34 (0.69–2.60)	0.39	1.84 (1.07–3.19)	0.03
Tumour necrosis (absent versus present)	1.28 (0.96–1.71)	0.09	1.46 (1.17–1.83)	0.001	1.10 (0.81–1.50)	0.53	2.46 (1.48–4.09)	0.001
Five-CpG-based classifier (low versus high risk)	4.10 (2.05–8.19)	<0.001	3.73 (2.28–6.09)	<0.001	3.36 (1.78–6.34)	<0.001	1.80 (1.11–2.93)	0.02

CI, confidence interval; HR, hazard ratio; MCHC, multiple clinical centres in China; SYSU, Sun Yat-sen University; TCGA, The Cancer Genome Atlas; UTSW, University of Texas Southwestern Medical Center at Dallas.

Tumour size was not included in the multivariate analysis due to colinearity with pathologic T stage.

^*^pN was not included in the multivariate analysis in TCGA set, because pN (N0 versus N1) was not a prognostic factor (*P*-value=0.21) in univariate Cox regression analysis and the nodal involvement status of 161 patients (54% of the total of 298 patients) was not available in this set.
