# Supplementary material for: A CpG-methylation-based assay to predict survival in clear cell renal cell carcinoma
Source: Nat Commun. 2015 Oct 30;6:8699. doi: 10.1038/ncomms9699 (PMC4846314; doi:10.1038/ncomms9699)
Supplement: Supplementary Information — Supplementary Figures 1-6 and Supplementary Tables 1-7 [file ncomms9699-s1.pdf]

Supplementary Figures

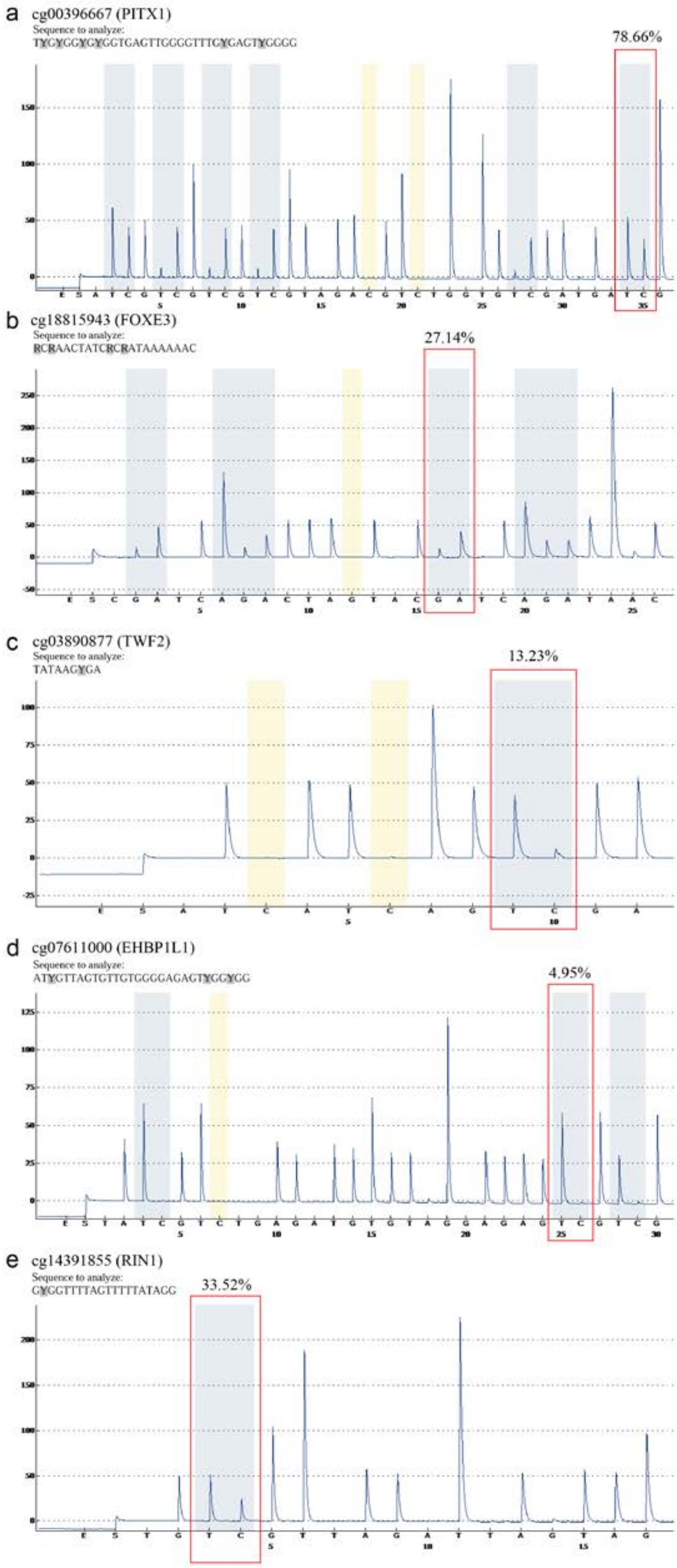

Supplementary Figure 1: Representative pyrograms for the five CpGs methylation analysis. Representative pyrograms for the five CpGs — (a) cg00396667 (PITX1), (b) cg18815943 (FOXE3), (c) cg03890877 (TWF2), (d) cg07611000 (EHBP1L1), and (e) cg14391855 (RIN1).

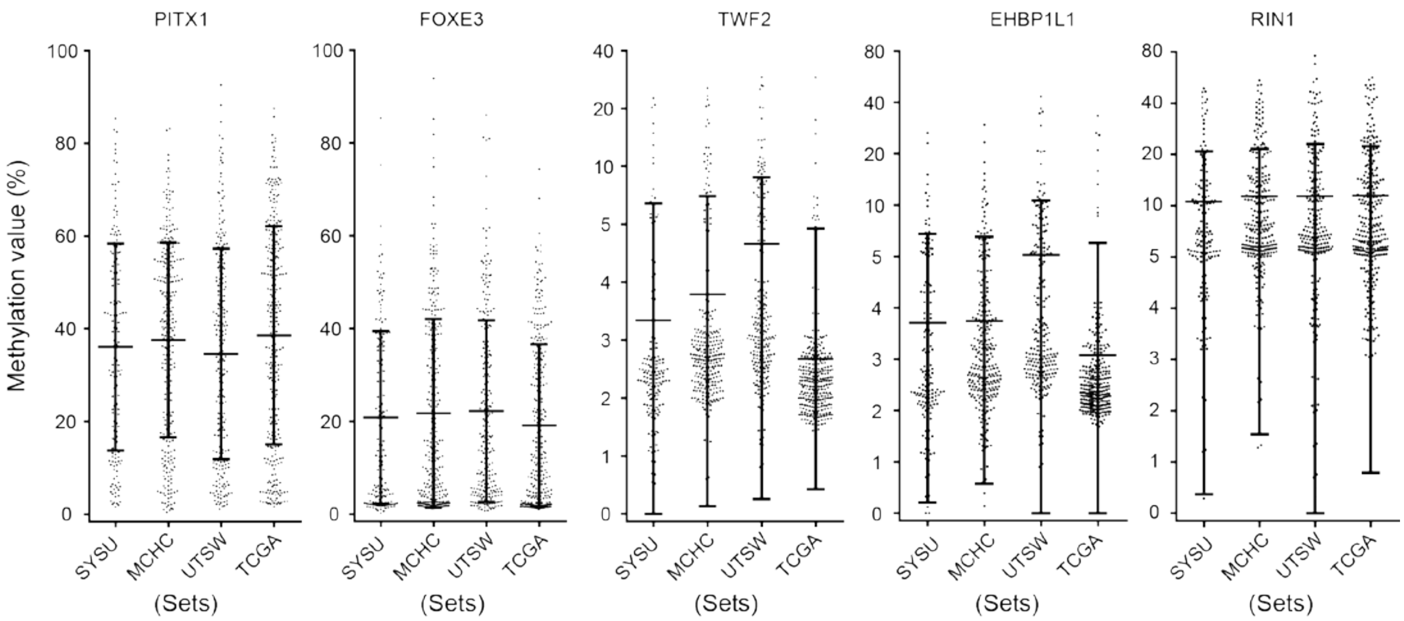

Supplementary Figure 2: Scatter dot plot depicting the methylation of the five CpG sites in the four patient sets. Methylation levels of the five CpG sites in the four patient sets. Mean value ± standard deviation (SD, black error bars) are reported.

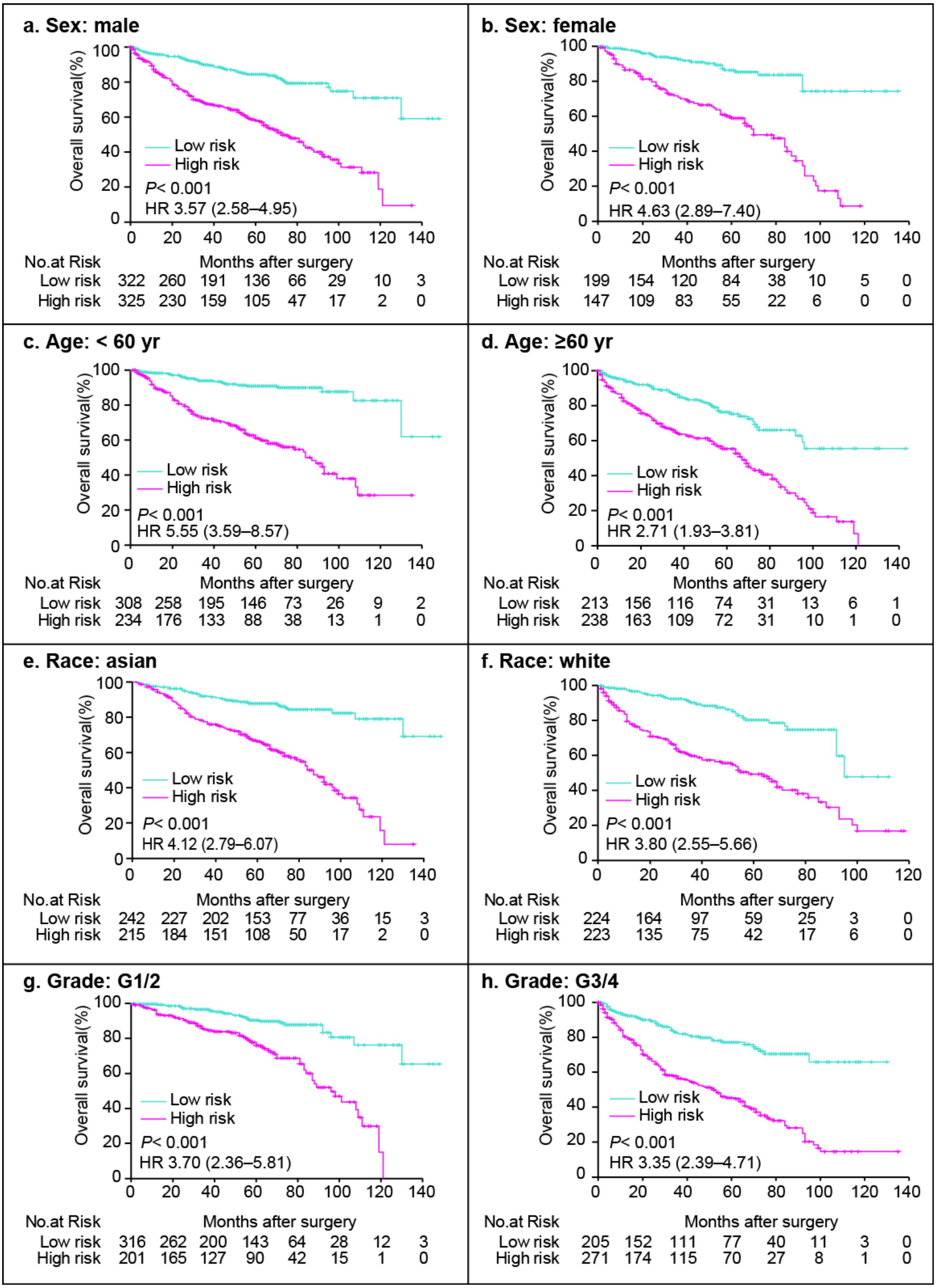
a. Sex: male
Overall survival(%)
Low risk
High risk
P< 0.001
HR 3.57 (2.58–4.95)
Months after surgery
No.at Risk
Low risk 322 260 191 136 66 29 10 3
High risk 325 230 159 105 47 17 2 0
b. Sex: female
Overall survival(%)
Low risk
High risk
P< 0.001
HR 4.63 (2.89–7.40)
Months after surgery
No.at Risk
Low risk 199 154 120 84 38 10 5 0
High risk 147 109 83 55 22 6 0 0
c. Age: < 60 yr
Overall survival(%)
Low risk
High risk
P< 0.001
HR 5.55 (3.59–8.57)
Months after surgery
No.at Risk
Low risk 308 258 195 146 73 26 9 2
High risk 234 176 133 88 38 13 1 0
d. Age: ≥60 yr
Overall survival(%)
Low risk
High risk
P< 0.001
HR 2.71 (1.93–3.81)
Months after surgery
No.at Risk
Low risk 213 156 116 74 31 13 6 1
High risk 238 163 109 72 31 10 1 0
e. Race: asian
Overall survival(%)
Low risk
High risk
P< 0.001
HR 4.12 (2.79–6.07)
Months after surgery
No.at Risk
Low risk 242 227 202 153 77 36 15 3
High risk 215 184 151 108 50 17 2 0
f. Race: white
Overall survival(%)
Low risk
High risk
P< 0.001
HR 3.80 (2.55–5.66)
Months after surgery
No.at Risk
Low risk 224 164 97 59 25 3 0
High risk 223 135 75 42 17 6 0
g. Grade: G1/2
Overall survival(%)
Low risk
High risk
P< 0.001
HR 3.70 (2.36–5.81)
Months after surgery
No.at Risk
Low risk 316 262 200 143 64 28 12 3
High risk 201 165 127 90 42 15 1 0
h. Grade: G3/4
Overall survival(%)
Low risk
High risk
P< 0.001
HR 3.35 (2.39–4.71)
Months after surgery
No.at Risk
Low risk 205 152 111 77 40 11 3 0
High risk 271 174 115 70 27 8 1 0

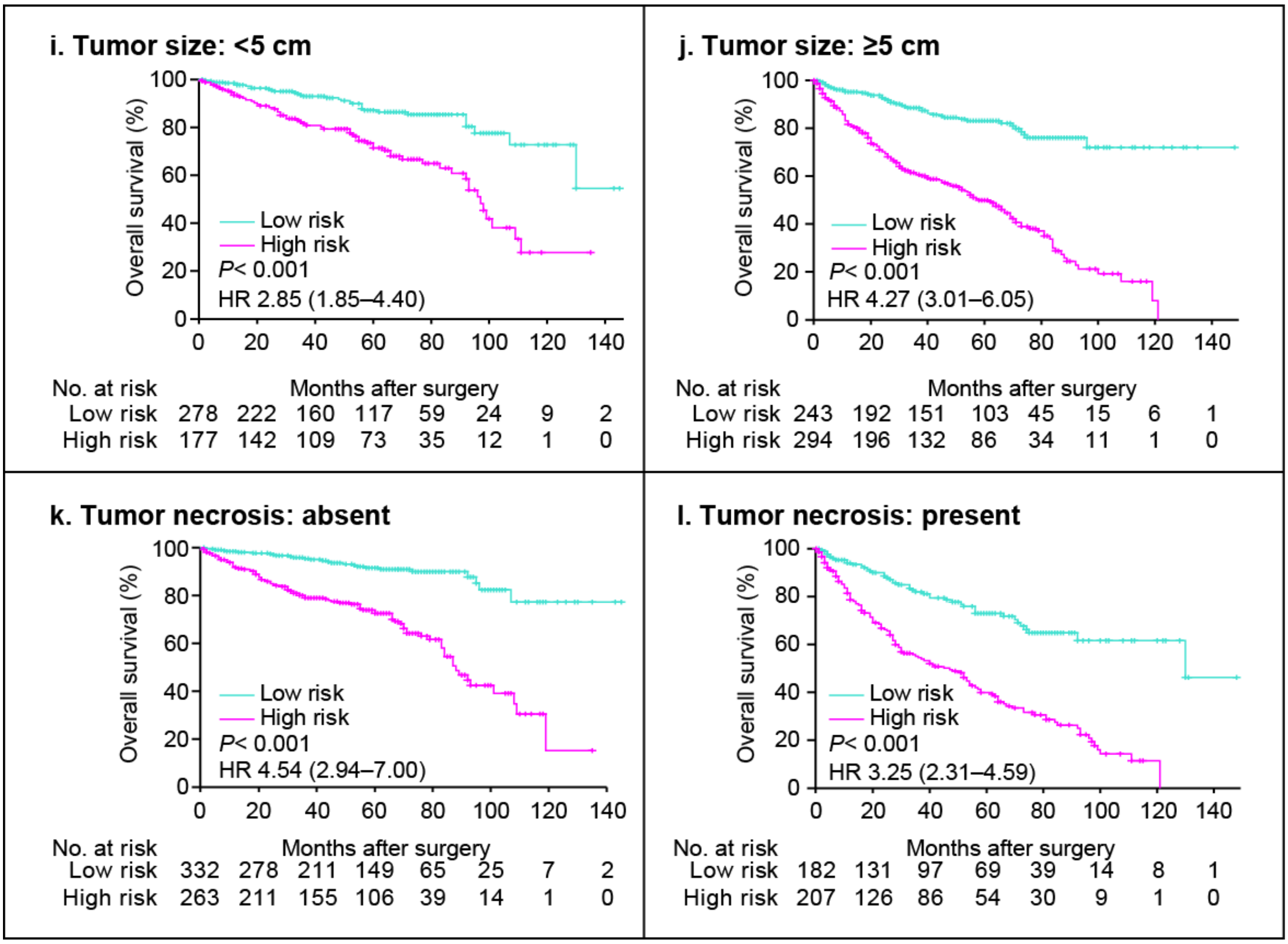

Supplementary Figure 3: Kaplan-Meier survival analysis for all 993 patients, according to the five-CpG-based classifier stratified by clinicopathological risk factors.

(a, b) sex, (c, d) age, (e, f) race, (g, h) Fuhrman grade, (i, j) Tumor size, and (k, l) Tumor necrosis. We calculated P values using the log-rank test.

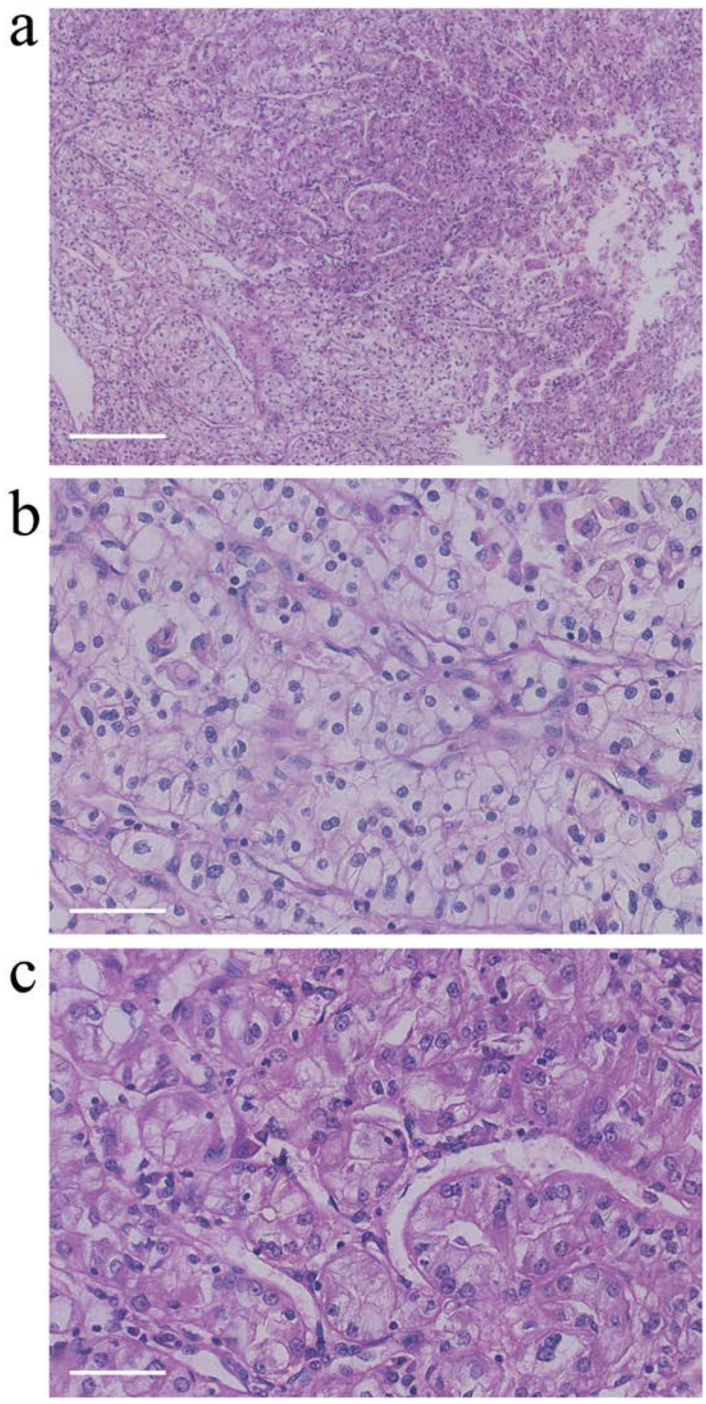

Supplementary Figure 4: Representative slides overview (H&E staining) of three different morphological regions from patient 3.
(a) Region 1: Clear cell renal cell carcinoma, showing nests of epithelial cells with clear cytoplasm and a distinct cell membrane, a small proportion of cells with eosinophilic cytoplasm. The upper of the picture was showing tumor regions with Grade 3, the lower of the picture was showing tumor regions with Grade 2. (H&E staining, 100×; Scale bar, 200 μm)
(b) Region 2: Clear cell renal cell carcinoma, Grade 2, with finely granular chromatin and small or ambiguous nucleoli. (H&E staining, 400×; Scale bar, 50 μm)
(c) Region 3: Clear cell renal cell carcinoma, Grade 3. When compare to Grade 2, the nuclei are larger, with nucleoli irregularity, obvious nucleoli. The nuclei pleomorphic are hard to see in this case. (H&E staining, 400×; Scale bar, 50 μm).

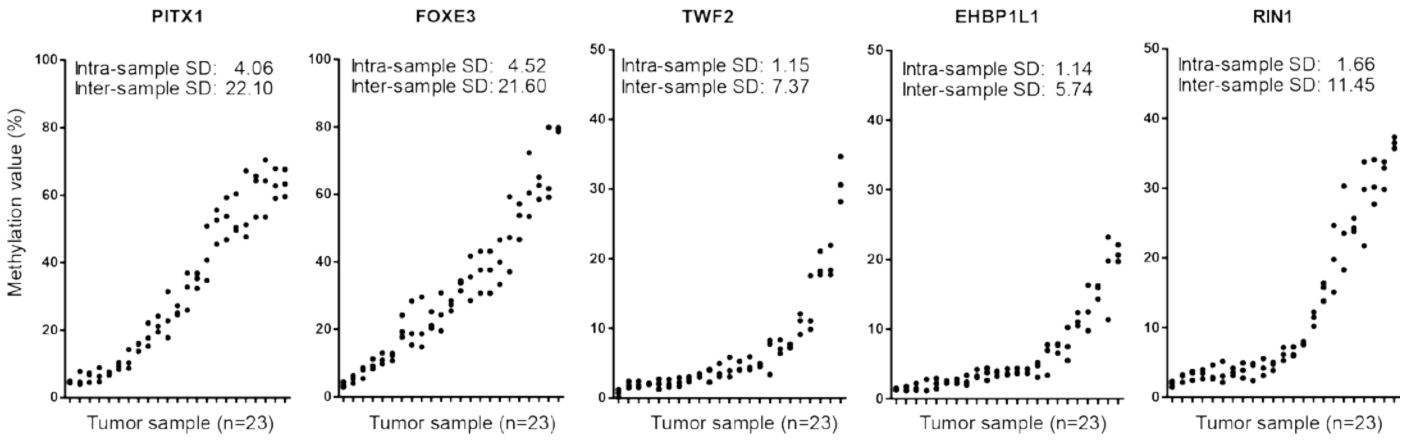

Supplementary Figure 5: Intra- and inter-sample variability of methylation at the five CpG sites. Methylation levels of the five CpGs — (a) cg00396667 (PITX1), (b) cg18815943 (FOXE3), (c) cg03890877 (TWF2), (d) cg07611000 (EHBP1L1), and (e) cg14391855 (RIN1) detected by pyrosequencing in three different regions within the tumors of 23 patients with ccRCC.

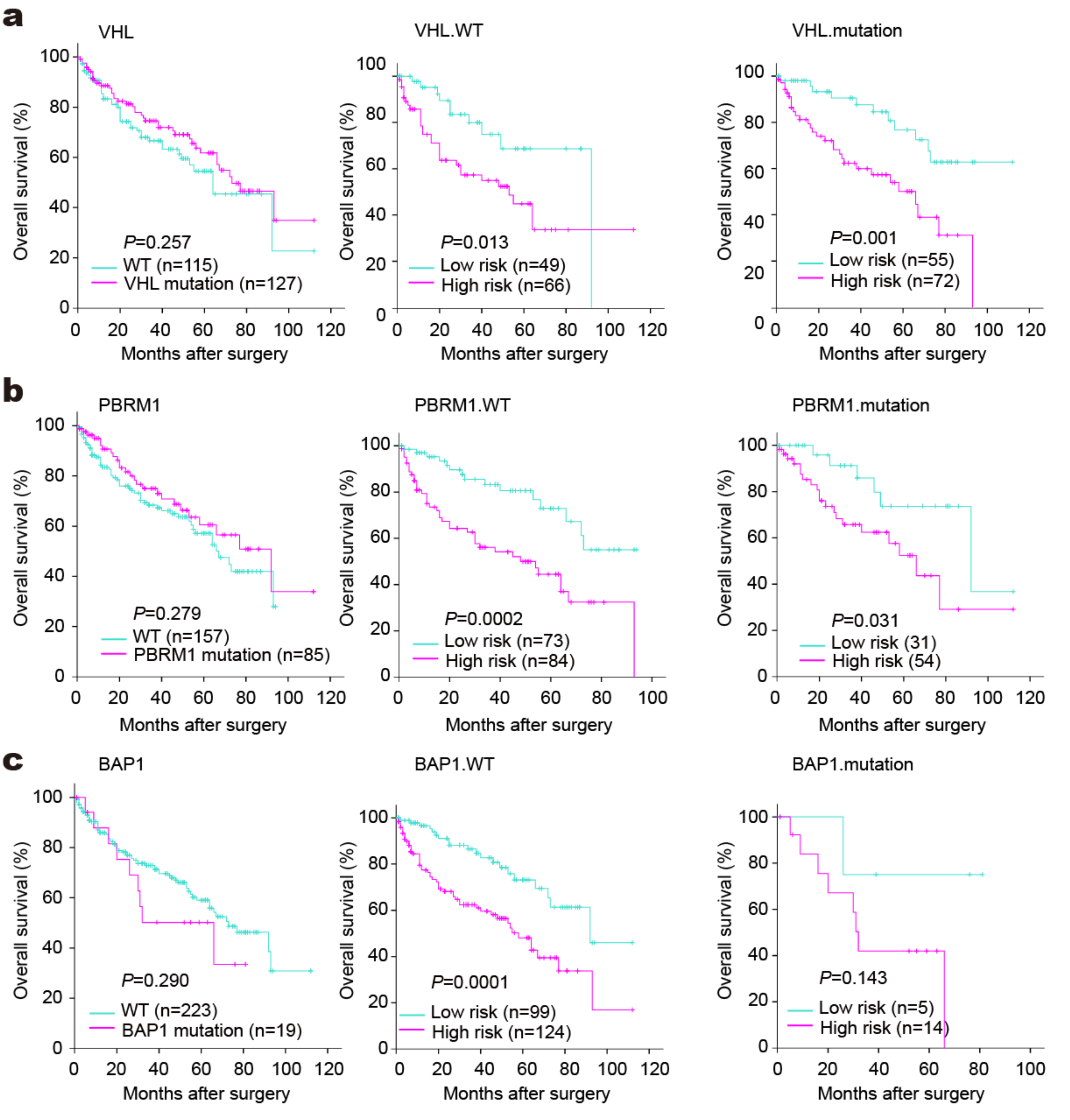

Supplementary Figure 6: Kaplan-Meier curves of overall survival for the indicated groups for 242 patients from the TCGA cohort.

Of the 298 patients from TCGA we analyzed in this study, somatic mutation data was available for 242. For the 242 patients, Kaplan-Meier survival analysis showed that *VHL*, *PBRM1*, and *BAP1* gene mutations did not correlate significantly with patients' overall survival (all P>0.05; figure 2, left panel). The five-CpG-based classifier successfully categorized patients into high-risk and low-risk groups with significant differences of clinical outcome in subsets of *VHL* wild type (*P*=0.013), *VHL* mutation (*P*=0.001), *PBRM1* wild type (*P*=0.0002), *PBRM1* mutation (*P*=0.031), and *BAP1* wild type (*P*=0.0001). In *BAP1* mutation subset, although not significant, there is still a trend to different outcomes between high-risk and low-risk groups (*P*=0.143). The small sample size of 19 patients might explain the negative results in the *BAP1* mutation subset. P values were calculated using the log-rank test.

Supplementary Tables

Supplementary Table 1: Clinical characteristics and gene mutation status (*VHL*, *BAP1*, and *PBRM1*) for 46 ccRCC patients from the discovery set.

ID	Sex	Site	pT	pN	M	Clinical stage	VHL mutation*	BAP1 mutation*	PBRM1 mutation*
2014ccRCC01	male	left	T1	N0	M0	I	NS	0	0
2014ccRCC02	male	right	T1	N0	M0	I	MS	0	FS
2014ccRCC03	male	left	T1	N0	M0	I	0	0	0
2014ccRCC04	female	left	T1	N0	M0	I	0	0	0
2014ccRCC05	female	left	T2	N0	M0	II	0	0	0
2014ccRCC06	male	right	T1	N1	M0	III	NS	0	FS
2014ccRCC07	male	right	T1	N0	M0	I	FS	0	0
2014ccRCC08	male	right	T1	N0	M0	I	0	0	0
2014ccRCC09	male	right	T1	N0	M0	I	FS	0	0
2014ccRCC10	male	right	T2	N0	M0	II	0	0	0
2014ccRCC11	female	right	T3	N0	M0	III	MS	FS	FS
2014ccRCC12	male	right	T1	N0	M0	I	FS	0	0
2014ccRCC13	male	right	T3	N1	M0	III	FS	0	0
2014ccRCC14	male	left	T2	N1	M0	III	IFD	0	0
2014ccRCC15	male	left	T2	N1	M1	IV	0	MS	0
2014ccRCC16	female	left	T3	N0	M0	III	0	0	0
2014ccRCC17	male	left	T1	N0	M0	I	FS	0	0
2014ccRCC18	female	right	T1	N0	M0	I	MS	0	FS
2014ccRCC19	female	left	T2	N0	M0	II	0	0	0
2014ccRCC20	female	right	T1	N0	M0	I	MS	0	FS
2014ccRCC21	female	left	T1	N0	M0	I	NS	0	FS
2014ccRCC22	male	right	T2	N0	M0	II	FS	0	0
2014ccRCC23	female	right	T1	N0	M0	I	MS	0	FS
2014ccRCC24	male	left	T1	N0	M0	I	0	0	0
2014ccRCC25	female	right	T1	N0	M0	I	0	0	NS
2014ccRCC26	male	left	T1	N0	M0	I	FS	0	0
2014ccRCC27	male	left	T1	N1	M0	III	NS	0	0
2014ccRCC28	male	left	T1	N0	M0	I	FS	0	0
2014ccRCC29	female	left	T2	N0	M0	II	FS	0	0
2014ccRCC30	male	right	T1	N0	M0	I	0	0	0
2014ccRCC31	male	right	T1	N0	M0	I	NS	0	0
2014ccRCC32	male	left	T3	N0	M0	III	0	0	NS
2014ccRCC33	male	left	T4	N0	M0	IV	SS	MS	0
2014ccRCC34	male	right	T3	N0	M0	III	0	0	0
2014ccRCC35	female	right	T2	N0	M0	II	0	0	0
2014ccRCC36	male	left	T1	N0	M0	I	0	0	NS
2014ccRCC37	female	left	T2	N0	M0	II	0	0	0
2014ccRCC38	male	left	T1	N0	M0	I	0	0	MS
2014ccRCC39	male	left	T1	N0	M0	I	FS	0	0
2014ccRCC40	male	left	T2	N0	M0	II	MS	0	FS
2014ccRCC41	female	right	T1	N0	M0	I	FS	0	0
2014ccRCC42	male	right	T2	N0	M0	II	0	0	0
2014ccRCC43	male	right	T1	N0	M0	I	0	0	0
2014ccRCC44	female	left	T4	N0	M1	IV	0	0	0
2014ccRCC45	male	left	T1	N0	M0	I	0	0	0
2014ccRCC46	female	left	T3	N0	M0	III	0	0	0

**VHL*, *PBRM1*, and *BAP1* mutation was analysis by using PCR amplification and bidirectional Sanger sequencing.
FS – frameshift. IFD – In-frame deletion. SS – Splice Site. NS – Nonsense. MS – Missense.

Supplementary Table 2: Primers for pyrosequencing.

ID	Chr	Position	Target Gene	UCSC Group	UCSC CpG Island	Primer sequence<br>F: forward; R: reverse; S: sequencing	Length (bp)	CpGs sequenced
cg00396667	5	134364387	PITX1	3'UTR	Island	F GGGGTTGAGTAGGTGTGA<br>R CTTACCAATACAACAACTAACC<br>S TGAGAAGGTGTGAGGT	153	7
cg03364683	4	169402298	DDX60L	TSS1500	Shore	F AATGTTTTTTAGTTTTGTTATTAAGTTTATTA<br>R CTTCCACCCTTTCCTTTTCTTA<br>S GTAATAAATGGAGAG	230	2
cg07166409	2	97534057	SEMA4C	5'UTR	Shore	F TTAGATTTATTATTATTTTTGAGTAATAGGAA<br>R CTCACCAAATTTCCCCTTTAA<br>S TTGAGTTTATTGAAA	247	1
cg03890877	3	52272690	TWF2	Body	Shore	F AATAGTTATTAGTGTTAGTGTATGAATGAT<br>R TAAAAAAAACTTCCTATTCTCTTTTACAA<br>S GTGAATGAATGAAAATAGATGTA	312	1
cg07611000	11	65342262	EHBP1L1	TSS1500	Island	F AGTGTAGGGGGTTGAGTATT<br>R CTTATTCCCCTCTCAATCTAT<br>S TAGATAATTGTAAAG	191	3
cg00026222	1	2144244			Island	F TGGAGGATAGAGGGTTTAGGT<br>R AACCCAACTACTCACAAAC<br>S GAGTATAGAGGGAGG	274	4
cg14391855	11	66104174	RIN1	TSS200	Shore	F AAGGTTTTTTGTAGTTTTATTAGTTATTTATA<br>R CCAAAAACAAATAAACAACTAACCAC<br>S GAGGGAAGTGGTTAA	138	1
cg13474998	3	53165031	RFT1	TSS1500	Shore	F ATTAGGAGTTTGGTATTTATTTAGTTAGG<br>R TCACAACCACAAACCCTCTA<br>S GGAAATAAGTTGTGT	238	1
cg12109838	4	103996978	NHEDC2	5'UTR	Shore	F GTTAGTTGTTTGGAAATTAAGGAT<br>R CTAAATACTAAAAATCATCTTATACTATTAAC<br>S ATAATTGAGTAGAAG	252	2
cg23497383	2	239140182	LOC151174<br>LOC643387	Body<br>TSS200	Island	F TTATAAGTTGAGGAGGGATTATAAT<br>R ACTACTAAAACTACAAAACCTT<br>S TAGTTAAGTAGTTTT	271	6
cg16518772	16	29819081	MAZ	Body	Island	F TTTTAGTTAGTTAAGGTTAGGTTA<br>R CCTACTAAATATATAACAAAACCTTCC<br>S TATAGAGTAGTTATGTGA	287	4
cg24888989	3	44803291	KIF15<br>KIAA1143	1stExon 5'UTR<br>TSS200	Island	F GTTTTTGTAGTTTATGTTTAGTAAGAATTTT<br>R TATCCAAACTCCAAATTCAA<br>S TTTATTTTTAGATTGTAT	254	7
cg00355909	16	66638320	CMTM3	5'UTR<br>1stExon<br>TSS1500	Island	F GTTTGTTTTTATTTAGAAGGGTTGTT<br>R TCACTACCTAAATCTTTACTATTT<br>S GAAGGGTTGTTTATA	118	5

cg23983315	7	73021609	MLXIPL	Body	Island	F GGGGAGGGTTAGGGAGGT	291	5
						R AAAATTAAACACCTAATCCCTTAC		
						S TAGGGAGGTTTTAGA		
cg05241461	19	22816980	ZNF492	TSS200	Shore	F TAGTTGAGTGTTTGATTGGAT	200	3
						R CTACTATATACTTAACTCAACTCAAAC		
						S AGAAGATGTAATTAA		
cg17557540	6	31166174	HCG27	Body	Shore	F GATTTATTAGGAGTAGAAGATTATTTTA	177	1
						R CAATCTAAATAACAAACCAAAACTATCT		
						S AAAGAAAGGGTGGGA		
cg18815943	1	47882314	FOXE3	1stExon	Island	F GTTAGGGAGGGAGTTAGAGGA	261	36
						R AAATTATAACCAATACTATTCTACCACTT		
						S AACCAATACTATTCTACCACTTAC		
cg02231066	3	52567130	NT5DC2	Body	Island	F GAGGTGTGTTTGGGAAGTTTA	135	9
						R CCCTTATTCTCCACCAACCCTTAAT		
						S ATTAAAACCAAAAAATACTATAAAC		

Supplementary Table 3: Univariate association of 18 CpGs with overall survival in the SYSU set.

ID	HR	95% CI		*P* value
		Lower	Upper	
cg00396667 (PITX1)	1.02	1.01	1.04	0.001
cg03364683	0.96	0.91	1.00	0.06
cg07166409	0.97	0.93	1.01	0.16
cg03890877 (TWF2)	0.76	0.60	0.96	0.02
cg07611000 (EHBP1L1)	0.82	0.70	0.97	0.02
cg00026222	0.97	0.92	1.02	0.29
cg14391855 (RIN1)	0.92	0.87	0.98	0.006
cg13474998	0.98	0.95	1.01	0.19
cg12109838	0.98	0.96	1.01	0.21
cg23497383	1.02	1.00	1.03	0.01
cg16518772	0.99	0.96	1.02	0.44
cg24888989	0.75	0.61	0.94	0.01
cg00355909	0.98	0.95	1.01	0.15
cg23983315	0.94	0.90	0.99	0.02
cg05241461	1.01	1.00	1.02	0.12
cg17557540	0.97	0.88	1.06	0.49
cg18815943 (FOXE3)	1.02	1.01	1.04	0.001
cg02231066	0.95	0.87	1.03	0.2

Supplementary Table 4: Univariate association of the five-CpG-based classifier with overall survival in the four sets.

Parameters	SYSU set		MCHC set		UTSW set		TCGA set	
	**HR (95%CI)**	***P* value**	**HR (95%CI)**	***P* value**	**HR (95%CI)**	***P* value**	**HR (95%CI)**	***P* value**
Sex (male vs female)	0.96 (0.51–1.78)	0.89	0.94 (0.59–1.49)	0.79	0.80 (0.47–1.35)	0.40	0.93 (0.61–1.43)	0.75
Age (younger than 60 years vs 60 years or older)	1.20 (0.68–2.13)	0.53	2.06 (1.34–3.16)	0.001	1.87 (1.11–3.16)	0.02	1.90 (1.21–2.97)	0.005
pT (T1/2 vs T3/4)	3.81 (2.08–6.99)	<0.001	2.91 (1.83–4.64)	<0.001	5.20 (3.03–8.92)	<0.001	3.78 (2.44–5.83)	<0.001
pN (N0 vs N1)	4.75 (2.33–9.69)	<0.001	6.68 (3.72–12.02)	<0.001	7.49 (3.89–14.43)	<0.001	1.93 (0.69-5.42)	0.21
M (M0 vs M1)	8.23 (2.44–27.71)	0.001	2.88 (1.16–7.15)	0.02	5.54 (3.03–10.12)	<0.001	4.59 (3.03–6.95)	<0.001
Grade (G1/2 vs G3/4)	2.72 (1.53–4.83)	0.001	2.35 (1.51–3.65)	<0.001	3.34 (1.94–5.75)	<0.001	3.36 (2.01–5.63)	<0.001
Tumor size (less than 5cm vs 5cm or greater)	1.45 (1.06–1.98)	0.02	1.34 (1.07–1.67)	0.009	1.50 (1.15–1.94)	0.003	1.64 (1.29–2.09)	<0.001
Tumor necrosis (absent vs present)	1.40 (1.06–1.85)	0.02	1.49 (1.20–1.84)	<0.001	1.70 (1.31–2.21)	<0.001	1.92 (1.51–2.45)	<0.001
Five-CpG-based classifier (low vs high risk)	4.27 (2.18–8.37)	<0.001	3.88 (2.41–6.27)	<0.001	4.82 (2.60–8.95)	<0.001	2.96 (1.86–4.72)	<0.001

Supplementary Table 5: Hazard ratio of overall mortality according to the five-CpG-based classifier in different age subgroups stratified by clinical parameters.

Subgroup	Age<60 yr (n=542)				Age≥60yr (n=451)			
	HR	95%CI		*P* value	HR	95%CI		*P* value
		Lower	Upper			Lower	Upper	
Sex								
Male	4.84	2.86	8.20	<0.001	2.42	1.60	3.67	<0.001
Female	6.73	3.10	14.58	<0.001	3.16	1.74	5.74	<0.001
Race								
White	6.50	2.98	14.15	<0.001	2.53	1.59	4.03	<0.001
Asian	5.52	3.11	9.79	<0.001	2.84	1.67	4.82	<0.001
Grade								
G1/2	5.94	2.70	13.09	<0.001	2.45	1.41	4.25	0.001
G3/4	4.63	2.71	7.89	<0.001	2.35	1.51	3.64	<0.001
Stage (clinical)								
I/II	5.01	2.60	9.68	<0.001	2.19	1.38	3.48	0.001
III/IV	3.64	2.02	6.55	<0.001	3.02	1.80	5.04	<0.001
Tumor necrosis								
Absent	8.09	3.79	17.30	<0.001	2.71	1.59	4.65	<0.001
Present	4.19	2.42	7.23	<0.001	2.48	1.59	3.84	<0.001
Tumor size								
<5cm	4.19	1.93	9.10	<0.001	2.07	1.22	3.49	0.007
≥5cm	5.61	3.30	9.53	<0.001	2.96	1.87	4.70	<0.001
All patients	5.55	3.59	8.57	<0.001	2.71	1.93	3.81	<0.001

Supplementary Table 6: The stage, size, grade, and necrosis (SSIGN) scoring algorithm.

**Parameter**	**Score points**
Pathologic tumor category	
pT1	0
pT2	1
pT3a	2
pT3b	2
pT3c	2
pT4	0
Nodal status	
NX	0
pN0	0
pN1	2
pN2	2
Metastasis category	
M0	0
M1	4
Tumor size	
<5 cm	0
≥5 cm	2
Grade	
1	0
2	0
3	1
4	3
Tumor necrosis	
Absent	0
Present	2

Supplementary Table 7: Summary of relevant finding of multiregional analysis for twenty three ccRCC patients.

Patient	Sex	Grade	TNM	Region	five-CpG-based classifier			PITX1 methylation		FOXE3 methylation		TWF2 methylation		EHBP1L1 methylation		RIN1 methylation	
				Sampled	stratification	risk score	CV (%)	value	CV (%)	value	CV (%)	value	CV (%)	value	CV (%)	value	CV (%)
Patient 1	F	G2	T1N0M0	R1	high risk	0.076	11.96	46.82	11.76	24.30	22.68	1.53	23.15	2.67	24.44	7.55	3.31
				R2	high risk	0.089		53.74		30.78		1.87		4.42		8.02	
				R3	high risk	0.096		59.33		19.54		2.42		3.78		7.61	
Patient 2	F	G2	T1N0M0	R1	***high risk***	-0.090	14.80	22.08	18.96	10.66	9.50	4.98	26.18	1.22	32.24	3.84	13.68
				R2	***low risk***	-0.117		17.65		12.86		3.46		2.23		4.79	
				R3	***low risk***	-0.118		15.23		12.22		3.08		1.46		5.01	
Patient 3	F	G3	T2N0M0	R1	low risk	-0.180	8.95	4.48	24.28	28.56	18.64	1.27	36.64	3.11	25.50	7.21	10.80
				R2	low risk	-0.150		7.27		35.54		2.04		4.79		5.93	
				R3	low risk	-0.164		6.86		41.70		2.75		5.22		6.11	
Patient 4	M	G4	T4N1M0	R1	high risk	0.366	3.57	59.59	6.36	62.61	5.36	4.39	20.91	4.31	8.67	1.46	21.75
				R2	high risk	0.386		63.32		65.12		4.05		4.22		2.28	
				R3	high risk	0.361		67.66		58.53		5.93		3.66		1.96	
Patient 5	F	G3	T1N0M0	R1	low risk	-0.647	8.80	27.25	5.48	19.29	16.50	11.11	13.88	15.97	6.62	10.18	9.17
				R2	low risk	-0.737		25.17		24.18		12.09		16.22		12.23	
				R3	low risk	-0.626		24.57		17.73		9.15		14.33		11.46	
Patient 6	M	G2	T3N0M0	R1	high risk	0.154	12.23	35.32	6.59	37.63	16.74	2.16	6.58	2.45	13.31	3.48	19.88
				R2	high risk	0.176		32.36		43.11		1.96		2.87		2.64	
				R3	high risk	0.138		36.88		30.70		1.91		2.21		3.96	
Patient 7	M	G3	T3N0M0	R1	low risk	-0.195	5.66	8.86	31.28	5.45	22.62	2.27	29.74	2.43	8.07	5.56	28.57
				R2	low risk	-0.186		4.74		7.88		4.11		2.22		3.12	
				R3	low risk	-0.208		6.33		8.61		3.98		2.61		4.17	
Patient 8	M	G3	T4N0M1	R1	high risk	0.501	4.03	64.24	13.66	61.70	16.88	0.73	79.56	1.21	54.35	3.04	44.64
				R2	high risk	0.466		53.53		59.18		0.12		1.16		2.14	
				R3	high risk	0.500		70.47		79.89		1.21		2.81		5.13	
Patient 9	M	G1	T1N0M0	R1	low risk	-0.626	8.49	67.22	18.83	47.18	23.30	7.75	41.53	6.95	38.73	29.85	21.64
				R2	low risk	-0.650		47.63		59.33		3.38		3.38		33.81	
				R3	low risk	-0.551		51.24		37.06		8.27		7.78		21.73	
Patient 10	M	G2	T1N0M0	R1	low risk	-0.672	10.59	60.35	11.25	46.59	10.29	7.05	13.73	7.91	9.64	29.85	6.45
				R2	low risk	-0.796		49.51		53.73		6.38		6.58		33.81	
				R3	low risk	-0.824		50.43		57.18		8.34		7.69		32.91	
Patient 11	M	G2	T3N0M0	R1	low risk	-1.539	3.58	16.14	8.49	27.28	5.60	30.59	10.52	11.04	8.65	23.79	4.08
				R2	low risk	-1.651		15.86		28.43		34.65		10.49		24.25	
				R3	low risk	-1.578		13.77		25.43		28.17		12.39		25.71	
Patient 12	M	G4	T4N0M0	R1	high risk	0.198	10.98	34.7	19.37	46.53	16.55	1.69	27.10	3.37	27.80	2.77	28.09
				R2	high risk	0.166		40.72		39.91		2.92		2.40		3.88	
				R3	high risk	0.205		50.83		33.32		2.22		1.97		4.94	

Patient 13	M	G3	T1N0M0	R1	low risk	-0.152	18.21	45.46	10.17	20.33	11.76	1.49	24.46	3.71	10.49	13.80	8.79
				R2	low risk	-0.147		55.59		25.19		1.77		4.39		15.76	
				R3	low risk	-0.202		52.65		21.10		2.39		3.65		16.38	
Patient 14	M	G2	T1N0M0	R1	***low risk***	-0.166	30.66	21.17	11.04	9.12	15.87	7.70	3.68	1.33	10.91	3.51	20.70
				R2	***high risk***	-0.095		24.15		11.22		7.24		1.25		2.47	
				R3	***low risk***	-0.178		19.44		8.28		7.22		1.54		3.72	
Patient 15	F	G3	T2N1M0	R1	low risk	-0.174	17.90	6.72	6.22	34.35	4.67	3.48	8.51	3.21	11.21	6.12	15.17
				R2	low risk	-0.209		7.35		31.41		3.04		3.77		7.13	
				R3	low risk	-0.146		7.59		33.72		2.99		4.01		5.26	
Patient 16	M	G2	T1N0M0	R1	low risk	-0.728	9.07	10.35	25.94	18.71	32.48	4.87	4.30	7.50	31.01	19.77	24.11
				R2	low risk	-0.782		14.29		28.38		4.95		5.49		24.67	
				R3	low risk	-0.652		8.68		15.36		4.56		10.28		15.10	
Patient 17	M	G2	T1N0M0	R1	low risk	-1.562	8.04	7.78	38.33	4.34	21.12	21.91	11.70	11.37	33.61	27.72	10.52
				R2	low risk	-1.817		4.52		3.33		17.70		19.81		34.11	
				R3	low risk	-1.782		3.93		2.89		18.37		23.20		30.15	
Patient 18	M	G2	T2N0M0	R1	low risk	-0.249	8.12	4.35	5.82	9.78	14.48	5.27	15.95	3.45	12.13	3.55	14.61
				R2	low risk	-0.214		4.74		10.86		4.11		4.31		3.12	
				R3	low risk	-0.221		4.87		12.97		3.98		3.60		4.17	
Patient 19	F	G3	T2N0M0	R1	high risk	0.501	6.28	64.24	10.85	78.59	0.78	2.71	12.89	1.16	25.96	3.21	20.78
				R2	high risk	0.456		53.53		79.79		3.07		1.21		2.14	
				R3	high risk	0.515		65.67		79.47		2.37		1.81		3.08	
Patient 20	F	G4	T3N1M0	R1	high risk	0.339	2.44	59.1	6.97	72.34	15.41	5.88	33.79	3.30	17.24	2.64	32.59
				R2	high risk	0.356		67.87		60.42		3.05		4.22		4.65	
				R3	high risk	0.349		62.74		53.43		3.93		3.07		2.86	
Patient 21	M	G2	T3N0M0	R1	low risk	-0.982	7.27	22.78	28.70	14.74	36.64	11.09	32.19	9.79	25.47	23.55	24.99
				R2	low risk	-1.098		17.83		29.58		17.56		16.31		18.30	
				R3	low risk	-1.129		31.45		18.65		9.87		12.49		30.29	
Patient 22	M	G2	T2N1M0	R1	high risk	0.158	25.79	25.89	17.51	37.63	16.74	1.59	26.64	1.44	34.42	2.39	34.30
				R2	high risk	0.099		32.78		43.11		2.69		2.95		4.60	
				R3	high risk	0.110		36.94		30.70		1.99		2.19		4.85	
Patient 23	F	G1	T1N0M0	R1	low risk	-1.963	3.10	9.45	10.63	5.33	21.86	21.08	9.57	19.73	5.75	37.32	2.19
				R2	low risk	-1.845		8.39		6.23		17.71		20.67		35.72	
				R3	low risk	-1.903		10.39		3.98		18.21		22.11		36.51	
**Average**							**10.46**		**15.15**		**16.37**		**22.31**		**20.71**		**18.30**
